# Combination therapies enhance immunoregulatory properties of MIAMI cells

**DOI:** 10.1186/s13287-019-1515-3

**Published:** 2019-12-18

**Authors:** Fiorella Rossi, Hunter Noren, Leonor Sarria, Paul C. Schiller, Lubov Nathanson, Vladimir Beljanski

**Affiliations:** 1 Cell Therapy Institute, Dr. Kiran C. Patel College of Allopathic Medicine, Nova Southeastern University, 3200 South University Drive, Fort Lauderdale, Davie, FL 33328 USA; 2 Institute for Neuroimmune Medicine, Dr. Kiran C. Patel College of Osteopathic Medicine, Nova Southeastern University, Davie, FL USA; 3 Department of Orthopaedics, University of Miami Miller School of Medicine, Miami, FL USA; 4 Prime Cell Biomedical Inc., Miami, FL USA

**Keywords:** Mesenchymal stromal cells, Immunoregulation, Autophagy, Tamoxifen, Combination therapies, Chloroquine, PD-L1, IDO

## Abstract

**Background:**

Mesenchymal stromal cells (MSCs), adult stromal cells most commonly isolated from bone marrow (BM), are being increasingly utilized in various therapeutic applications including tissue repair via immunomodulation, which is recognized as one of their most relevant mechanism of action. The promise of MSC-based therapies is somewhat hindered by their apparent modest clinical benefits, highlighting the need for approaches that would increase the efficacy of such therapies. Manipulation of cellular stress-response mechanism(s) such as autophagy, a catabolic stress-response mechanism, with small molecules prior to or during MSC injection could improve MSCs’ therapeutic efficacy. Unfortunately, limited information exists on how manipulation of autophagy affects MSCs’ response to inflammation and subsequent immunoregulatory properties.

**Methods:**

In this study, we exposed BM-MSC precursor cells, “marrow-isolated adult multilineage inducible” (MIAMI) cells, to autophagy modulators tamoxifen (TX) or chloroquine (CQ), together with IFN-γ. Exposed cells then underwent RNA sequencing (RNAseq) to determine the effects of TX or CQ co-treatments on cellular response to IFN**-γ** at a molecular level. Furthermore, we evaluated their immunoregulatory capacity using activated CD4+ T cells by analyzing T cell activation marker CD25 and the percentage of proliferating T cells after co-culturing the cells with MIAMI cells treated or not with TX or CQ.

**Results:**

RNAseq data indicate that the co-treatments alter both mRNA and protein levels of key genes responsible for MSCs’ immune-regulatory properties. Interestingly, TX and CQ also altered some of the microRNAs targeting such key genes. In addition, while IFN-γ treatment alone increased the surface expression of PD-L1 and secretion of IDO, this increase was further enhanced with TX. An improvement in MIAMI cells’ ability to decrease the activation and proliferation of T cells was also observed with TX, and to a lesser extent, CQ co-treatments.

**Conclusion:**

Altogether, this work suggests that both TX and CQ have a potential to enhance MIAMI cells’ immunoregulatory properties. However, this enhancement is more pronounced with TX co-treatment.

## Background

Mesenchymal stromal cells (MSCs) are adult progenitor cells that can be differentiated into cell types of mesodermal origin, such as adipocytes, osteocytes, and chondrocytes [1]. Due to their immunoregulatory properties coupled with abundance and straightforward expansion protocols, MSCs have been identified as promising candidates for cellular therapy [2, 3]. The pleiotropic effects of MSCs allow for their use in a variety of therapeutic applications: MSCs’ immunomodulatory capacity is activated in response to a pro-inflammatory environment and can contribute to treatment of injury associated with, or caused by, excessive inflammation [4]. The therapeutic benefits of MSCs have been observed in a wide range of pre-clinical models of diseases caused by excessive inflammation such as graft-versus-host disease (GVHD), multiple sclerosis, rheumatoid arthritis, and sepsis [5]. Numerous other studies have also evaluated MSC-mediated immune modulation and established that administration of MSCs, either systemically or locally, induce T cell anergy, reduce the numbers of pro-inflammatory T helper 17 cells, and increase the numbers of immunosuppressive regulatory T cells capable of suppressing T-effector responses [6].

MSCs have many advantages for clinical use compared to other cell types (such as embryonic stem cells, ESCs) due to their adult origin and due to their ability to repopulate, differentiate, and cause immunoregulation similar to ESCs without the risk for teratoma formations [7]. Moreover, MSCs are free of ethical issues concerning their origin unlike to embryonic stem cells [8]. Unfortunately, there are still several factors that limit their applications; for example, MSCs undergo replicative senescence upon continuous passaging which has been shown to compromise their therapeutic properties [9]. Additionally, the use of MSCs in regenerative medicine requires multiple injections of a large number of cells in order to confer clinical benefits and MSCs injected have been showed to have low survival resulting in limited regeneration of damaged tissues [10]. One powerful tool to overcome these limitations and potentiate MSC immunoregulation involves modification of MSCs’ biology. These modifications could improve their multipotency and/or differentiation capacity, enhance the secretion of immunoregulatory molecules, and ultimately extend MSC survival post-injection.

Therapeutic properties of MSCs could be enhanced with small molecules—this is an attractive approach as small molecules can be used to target key properties of MSCs and properties of adjacent tissues simultaneously. Among “druggable” pathways, evidence points at a key role of autophagy in the maintenance and function of MSCs [11, 12]. Autophagy is an evolutionary conserved lysosomal catabolic pathway that plays a major role in maintaining cellular homeostasis (reviewed in [13]) which also contributes to the regulation of intrinsic, innate, and adaptive immunity, including regulation of immunomodulatory cytokine levels [1, 14–18]. Interestingly, autophagy in MSCs has also been associated with the regulation of stemness, cellular maintenance, and with the regulation of somatic reprogramming processes [19]. Because of such important roles, autophagy is an attractive pathway that, when manipulated with small molecules, could impact the therapeutic properties of MSCs. Unfortunately, little is known how manipulation of autophagy affects immunomodulatory properties of MSCs. There are several drugs that can be used to modulate autophagy; however, the use of tamoxifen (TX) and chloroquine (CQ) has some advantages since these drugs are Food and Drug Administration (FDA) approved and available to use for specific treatments. TX induces autophagy via multiple mechanisms, while CQ blocks autophagosome-lysosome fusion, thus inhibiting autophagy [20, 21].

To test the impact of these two autophagy modulators on MSCs’ immunoregulatory properties, we utilized MSC progenitor cells termed “marrow-isolated adult multilineage inducible” (MIAMI) cells which are isolated from the bone marrow of cadaveric donors using specific methodologies [22]. MIAMI cells are significantly more homogeneous, developmentally immature with proteome and secretome profiles that are somewhat distinct compared to bone marrow-isolated MSCs (BM-MSCs): they highly express telomerase reverse transcriptase and are capable of extensive expansion in vitro. The cells isolated from donors display consistently similar gene expression independent of age and gender and appear to have more proteins in common with human ESCs than do MSCs [23, 24], but without the potential of forming teratomas [25], since it was shown that they exhibit non-tumorigenic properties [22]. Because of these characteristics, MIAMI cells and their products are now being tested in various clinical applications including tissue regeneration [26, 27]. Even though MIAMI cells express several markers similar to ESCs, they do not possess total ability of self-renewal; however, they appear to respond to specific molecular signals in the appropriate environmental conditions to induce self-renewal [28]. This can be used to manipulate MIAMI cells to become more stable, maintain their pluripotency, and sustain their immunoregulatory properties for longer time periods. Therefore, we decided to utilize MIAMI cells in combination with TX or CQ.

## Material and methods

### Materials

Two MIAMI cell donors labeled 3515 and 4381 were obtained from Dr. Paul Schiller’s laboratory who isolated them from commercially available human BM-MSCs (Lonza, Walkersville, MD) [29]. Media for growth were DMEM Media - GlutaMAX™ (Thermo Fisher). Adipose-derived MSCs were purchased from Lonza and were grown in Mesenchymal Stem Cell Basal Medium combined with MSC growth kits (Lonza). Tamoxifen (TX) and IFN**-**γ were purchased from Sigma-Aldrich, and chloroquine (CQ) was purchased from VWR.

### Cell isolation and culture

Buffy coats were obtained from Continental Service Group Blood Bank (Fort Lauderdale) with K_2_EDTA as an anti-coagulant. Donor inclusion criteria were set at age 20–40 years with a BMI of 29 or less, non-smokers, and free of HIV, hepatitis B, hepatitis C, syphilis, West Nile virus, and ZIKA virus. Peripheral blood mononuclear cells (PBMCs) were isolated the same day as collection by diluting the buffy coat at 1:1 ratio with PBS (without Ca^2+^ and Mg^2+^) and overlaid on Ficoll-Paque PLUS density gradient media (GE Healthcare Life Sciences) then separated by gradient centrifugation. CD4+ T cells were isolated from the PBMCs by negative selection using human CD4+ T Cell Isolation Kit (Milteny Biotec). Isolated CD4+ T cells were cultured in RPMI Medium 1640 - GlutaMAX™-I (Gibco™), 10% heat-inactivated FBS (GE Healthcare), 1× Antibiotic-Antimycotic Solution (Corning), and 20 ng/ml IL-2 (Roche) in a 37 °C, 5% CO_2_ incubator. MIAMI cells from two different donors were used in experiments (labeled #3515 and #4381); the cells were cultured in DMEM-GlutaMAX™ (Gibco™), 3% FBS (GE Healthcare), 1× lipids [22], 1× Antibiotic-Antimycotic Solution (Corning), and 100 μM Ascorbic Acid 2-Phosphate (Sigma). MIAMI cells were grown in 10 ng/ml fibronectin (Sigma-Aldrich)-coated flasks at a density of 400 cells/cm^2^ at 3% O_2_/5% CO_2_/92% N_2_. To maintain the cells, 60% of old medium was replaced with fresh medium every 2–3 days. MIAMI cells were expanded at low density (up to 40% confluency) using Gibco™ Trypsin-EDTA (0.05%). Cells were maintained in culture and used at low passages (*p* < 8).

### Pharmacological modulation of autophagy

MIAMI cells were seeded in fibronectin-coated 6-well plates at a density of 1 × 10^5^ cells/well and treated with different concentration of TX and CQ. After 4 days of treatment, autophagy was assessed by immunoblotting for LC3B-II (Novus Biologicals, LLC). β-actin antibody (Abcam) was used to semi-quantify protein bands using densitometry analysis (ImageJ, NIH, USA). Autophagic vesicles were also assessed by Cyto-ID autophagy detection kit (Enzo Life Sciences, Inc.). Additionally, other molecules in the autophagy pathway were evaluated by immunoblotting: ATG5 (Cell signaling) and BECN1 antibody (Invitrogen).

### RNA sequencing

MIAMI cells were seeded in fibronectin-coated 6-well plates at a density of 1 × 10^5^ cells/well and treated with 500 U/ml IFN-γ alone, or in combination with 5 μM TX or 10 μM CQ for 4 days. Following the treatments, medium was aspirated from cells, and cells were detached from wells by RNAzol and stored at − 80 °C until RNA isolation. RNA was purified using RNAzol (Molecular Research Center) according to the manufacturer’s instructions. Fifty nanograms of total RNA was submitted to the NSU Genomic Core, and 150 base paired-end read sequencing was performed using Illumina NextSeq500. All procedures were performed according to the manufacturer’s instructions. Quality control assessment was done using Illumina RNAseq pipeline to estimate genomic coverage, percent alignment, and nucleotide quality. Raw sequencing data were transformed to fastq format. Raw reads were mapped to the reference human genome (the most recent build GRCh38) using GSNAP, MapSplice, HISAT2, and STAR software. Reads for each gene aligned by GSNAP, MapSplice, and HISAT2 were counted using HTSeq software. Alignment by STAR was run with the option “quantMode TranscriptomeSAM” that allowed counting of reads aligned to each gene. Raw counts from HTSeq and STAR were imported into Bioconductor/R package edgeR, normalized and tested for differential gene expression. This analysis was done separately for the files produced by each aligner. In each analysis, we selected genes that were expressed differentially based on the criteria of false discovery rate (FDR) less than 5% and fold change more than 2.0 to either direction. Genes that showed differential expression after analysis of the files from at least two aligners were selected for further analysis. A list of these genes was imported into the Ingenuity Pathway Analysis software (IPA, Qiagen) for pathway analysis and for analysis of upstream regulators. Gene set enrichment analysis was performed using Gene SeT AnaLysis Toolkit (WebGestalt) [30].

### NanoString assessment of gene expression

Validation of expression for selected genes involved in immune-regulation was performed using nCounter Elements technology (NanoString, Seattle, USA). nCounter Elements allows users to combine nCounter Elements General Purpose Reagents (GPRs) with unlabeled probes that target specific genes of interest (www.nanostring.com/elements/). One hundred nanograms of total RNA from each sample was used to hybridize with the nCounter Elements TagSet at 67 °C for 16 h. The TagSet consists of a reporter tag and capture tag that hybridize to the user-designed gene-specific probe A and probe B complex. After hybridization, the samples were washed and immobilized to a cartridge using the NanoString nCounter Prep Station. Cartridges were scanned in the nCounter Digital Analyzer at 280 fields of view for the high level of sensitivity. Positive NanoString spike-in controls and 5 highly invariant genes (SAR1B, YWHAB, ETFA, SPEN, and SEC24C) served as internal controls for normalization between samples. This produced 43 genes suitable for validation (Additional file 4).

### NanoString microRNA expression analysis

Total RNA was used for human NanoString nCounter microRNA (miRNA) assay (NanoString Technologies, Seattle, WA, USA) according to the manufacturer’s instructions using the nCounter Human miRNA Panel v3 that evaluates 800 miRNAs. Total RNA extracted from MIAMI cells was subjected to nCounter miRNA sample preparation according to the manufacturer’s instructions. The nCounter results were analyzed by the nSolver 3.0 software (NanoString Technologies, Seattle, WA, USA). We calculated a background level of expression using the geometric mean of the negative controls plus two SD. miRNAs that were expressed lower than background level were excluded from further analysis. After that, miRNA counts were normalized using the geometric mean of the top 100 miRNAs, according to the manufacturer’s protocol. Partek Genomic Suite (Partek, Inc.) v6 was used to evaluate differential expression of miRNA.

### miRNA mimic transfection

MIAMI cells were seeded at a density of 2 × 10^5^ cells/well in fibronectin-coated 6-well plates and transfected with 100 nM each miRNA mimics: hsa-miR-127-3p, hsa-miR-1303, and hsa-miR-100-5p, and miRNA negative control (300 nM, mirVana, Ambion) using StemFect RNA transfection kit (Stemgent) for 48 h. Total RNA was extracted and cDNA was synthetized using 500 ng of RNA by qScript™ SuperMix (Quanta bio), and gene expression was measured by qPCR using PerfeCta® SYBR® Green SuperMix, Low Rox in AriaMx qPCR System (Agilent Technologies).

### Apoptosis assay

1 × 10^5^ MIAMI cells were seeded in fibronectin-coated plates and treated with 5 μM TX or 10 μM CQ to determine the induction of cell death or apoptosis at day 4 post-treatment. Cells were stained with Annexin V-FITC (BD Bioscience) and 7-aminoactinomycin D (7-AAD, Biolegend) and analyzed by flow cytometry (BD LSRFortessa™ X-20, BD Bioscience).

### CD4+ T cell assays

CD4+ T cells (purity > 92%) from 8 different donors were cultured with 20 ng/ml of IL-2 (Roche) and activated using 30 μl of ImmunoCult™ Human CD3/CD28 T Cell Activator (STEMCELL Technologies) per 1 × 10^6^ cells/ml. 2 × 10^6^ CD4+ T cells were plated in the presence or absence of 1 × 10^5^ MIAMI cells. TX or CQ were also added to co-cultures of CD4+ T and MIAMI cells (20:1, respectively). After 3 days of co-culture, CD4+ T cells were collected and stained with Live/Dead-Aqua (Invitrogen), CD4-FITC (BD Bioscience), and activation marker CD25-PE (BD Bioscience) then analyzed by flow cytometry (BD LSRFortessa™ X-20, BD Bioscience) by gating only on live cells.

### CD4+ T cell proliferation assay

2 × 10^6^ CD4+ T cells were stained with 1 μM of CellTrace™ Carboxylfluorescein Succinimidyl Ester (CFSE) Cell Proliferation Kit for flow cytometry (Thermo Fisher SCIENTIFIC). CFSE-stained T cells were activated as outlined above and co-cultured with MIAMI cells at a ratio of 20:1 (CD4+ T to MIAMI cells). At day 3, additional 30 μl ImmunoCult™ Human CD3/CD28 T Cell Activator was added, and at day 4, CD4+ T cells were harvested and stained with the cell viability marker 7-AAD (Biolegend) and CD4-BUV395 (BD Bioscience) and assessed by flow cytometry for CFSE dilution on live CD4+ T cells.

### MIAMI cell supernatant assay

To assess the effect of paracrine factors secreted by MIAMI cells, 1 × 10^5^ MIAMI cells were seeded in 6-well fibronectin-coated plates and treated with 5 μM TX or 10 μM CQ in the presence of IFN-γ. As experimental controls the same concentration of TX, CQ and IFN-γ were added to cell media in plates without cells. After 4 days of incubation, supernatants from each treatment were collected and used to treat activated CD4+ T cells and their effect on CD4+ T cell proliferation was evaluated as previously described.

### Cytokine secretion assays

To assess the secretion of cytokines in co-culture assays, supernatants were collected from CD4+ T cell assays from different donors (*n* = 8) on day 3 after co-culture with/without MIAMI cells. IL-6 was measured by enzyme-linked immunosorbent assay (Human IL-6 ELISA MAX™ Set Deluxe, BioLegend).

### Statistical analysis

Statistical analyses were performed using GraphPad Prism (GraphPad Software Inc., La Jolla, CA, version 8). Data are expressed as means ± standard errors of the mean (S.E.M). One-way analysis of variance (one-way ANOVA) test was used to determine statistical significance between each treatment group, if present. *p* values ≤ 0.05 were considered statistically significant.

## Results

### CQ and TX alter IFN-γ-induced gene expression

**Fig. 1:**
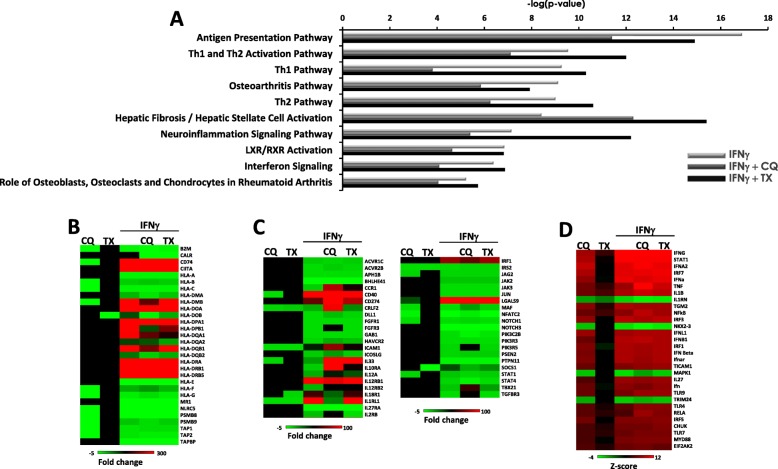
TX and CQ co-treatments alter mRNA levels of genes involved in response to inflammation. MIAMI cells were treated with 500 U/ml IFN-γ alone or in combination with 10 μM CQ or 5 μM TX. Total RNA was isolated and subjected to RNAseq. Sequencing data were processed as described in the “Material and methods” section. IPA gene expression analysis was performed to determine signaling pathways that are involved in MIAMI cell’s response to IFN-γ alone or in combination with TX or CQ (**a**). Representative heatmaps for genes belonging to antigen presentation (**b**) and Th1 and Th2 activation pathways (**c**) are shown. Upstream pathway analysis was performed to determine putative upstream regulators (**d**)

To determine how TX or CQ co-treatments affect transcriptional responses of MIAMI cells to inflammation stimulation, we performed RNA sequencing (RNAseq) of MIAMI cells after CQ or TX co-treatments. MIAMI cells were exposed for 4 days to either 500 units of IFN**-**γ alone or together with 5 μM TX or 10 μM CQ. At these doses and exposure time, TX or CQ did not cause apoptosis in MIAMI cells but did result in the accumulation of autophagosomes (Additional file 1: Figure S1). Upon completion of treatment, cells were flash-frozen; total RNA was extracted and subjected to RNAseq. The list of differentially expressed genes (fold changes > 2.0, false discovery rate < 0.05%) was uploaded into ingenuity pathway analysis (IPA), and the top differentially regulated genes and pathways were analyzed for their functions. Canonical pathway analysis using IPA software identified differential expression of genes belonging to the antigen presentation pathway in addition to various other pathways related to inflammation and IFN**-**γ signaling as the main functional categories (Fig. 1a). Interestingly, co-treatment with either CQ or TX altered statistical significance of the pathways (which is related to both the number of genes assigned to a pathway and the extent of their differential expression) compared to IFN**-**γ treatment alone. Co-treatment with CQ led to an even greater decrease in inflammation-related pathways upon IFN**-**γ exposure. The heatmaps on Fig. 1b, c illustrate changes in gene expression resulting from TX or CQ co-treatment. Importantly, co-treatments typically changed expression levels of various genes belonging to inflammation-related pathways, which in turn changed the contribution of the respective pathway to the overall response. This was more pronounced in cells co-treated with CQ compared to cells co-treated with TX. Importantly, co-treatments did not change which genes were differentially regulated; rather, they modulated the extent of differential expression. Next, we utilized IPA upstream regulator analysis which is based on pre-determined links between transcriptional responses of target genes and their regulators stored in the IPA database [31]. This analysis determined that co-treatments did not change the identity of (putative) upstream regulators or the nature of pro-inflammatory response, but rather, they “fine-tuned” how cells responded to IFN**-**γ (Fig. 1d). These data were further corroborated using functional gene set enrichment analysis (GSEA) tool WEB-based GEne SeT AnaLysis Toolkit (WebGestalt) which indicated no change in biological processes, cellular components, or molecular function when MIAMI cells were exposed to IFN-γ alone or also co-treated with TX or CQ (Additional file 2: Figure S2). We also determined pathway network connections between distinct treatments by examining top differentially regulated pathways and the number of common genes (Additional file 3: Figure S3). These results indicate that while top pathways are mostly preserved, some differences exist in the numbers of common genes. In addition, the significance of dendritic cell maturation pathway appears to be altered with TX and CQ treatments which can influence cell-cell interactions between MIAMI and T cells.

### TX or CQ co-treatments alter differential expression of immunomodulatory genes

**Fig. 2:**
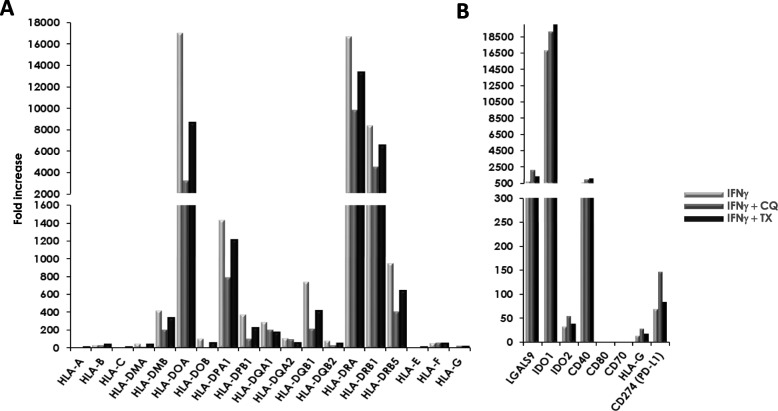
TX and CQ co-treatments change mRNA levels of HLA and immunomodulatory genes. MIAMI cells were treated with 500 U/ml IFN-γ with or without 10 μM CQ or 5 μM TX for 4 days. Fold changes in mRNA levels were assessed via RNAseq analysis. **a** Fold induction of mRNA levels of HLA genes. **b** Fold induction of immunomodulatory and co-stimulatory genes

Next, we examined how CQ or TX co-treatments affected IFN**-**γ-induced changes in expression of genes known to be responsible for the immunomodulatory properties of MSCs (Fig. 2). While there was very little effect on the expression of HLA-A, HLA-B, or HLA-C genes with either CQ or TX co-treatments, combining either CQ or TX with IFN**-**γ altered mRNA expression of MHC class II genes (Fig. 2a). This change was more pronounced for CQ compared to TX. For example, 40–80% decrease in mRNA expression was observed among HLA-DOA, HLA-DOB, HLA-DPB1, and HLA-DQB1 genes when cells were co-treated with CQ. A less pronounced decrease (20–40%) was observed among HLA-DMA, HLA-DMB, HLA-DPA1, HLA-DQB2, HLA-DRA, HLA-DRB1, and HLA-DRB5 when co-treated with TX. Furthermore, we also evaluated mRNA levels of several other genes that were previously associated with immunomodulatory properties of MSCs (Fig. 2b). While no mRNA increases in co-stimulatory CD70 or CD80 was observed with TX or CQ co-treatments, levels of immunomodulatory HLA-G and PD-L1 were increased, especially for CQ co-treatment. Altogether, these data indicate that both drugs have an effect on gene expression and can change how MSCs respond to inflammation on a molecular level.

We next confirmed RNAseq data by examining changes in mRNA levels for numerous HLA genes using the same samples that were used for RNAseq. Additionally, to confirm the effects of TX or CQ on gene expression in the presence of IFN**-**γ in different cells, we utilized another MIAMI cell donor and another MSC type, adipose-derived MSCs (Ad-MSCs). Cells were subjected to the same treatments as for RNAseq, and gene expression was assessed via NanoString technology. We found that TX or CQ co-treatments induce similar changes in gene expression in different MIAMI cell donors and in Ad-MSCs compared to the MIAMI cells used for RNAseq (Additional file 3: Figure S3). These results indicate that the two drugs may produce similar effects upon stimulation of inflammation in different MSCs.

### miRNAs contribute to TX- and CQ-mediated changes in gene expression

miRNAs are small RNAs that play a key role in the regulation of gene expression by controlling stability and translation of messenger RNAs (mRNAs) of protein-coding genes. Therefore, miRNAs modulate and orchestrate cellular pathways, including cell growth, differentiation, inflammation, apoptosis, and other pathways. To evaluate whether changes in miRNA expression contributed to observed changes in gene expression, we assessed 800 biologically relevant miRNAs using NanoString’s pre-built panels for human miRNA. mRNA levels were measured using the same total RNA isolates that were utilized previously for RNA sequencing. Both sets of data were subsequently uploaded to IPA and subjected to miRNA target filter analysis in order to examine miRNA-mRNA pairings based on IPA’s database of both predicted and literature-reported pairing relationships from TargetScan.

**Fig. 3:**
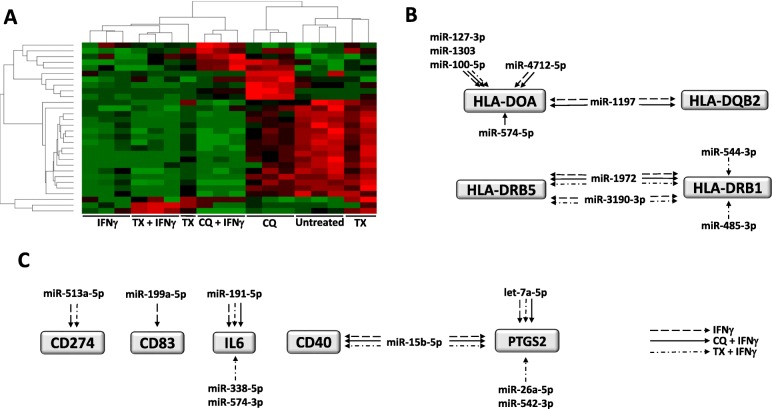
The effect of IFN-γ and TX or CQ co-treatments on miRNA levels. NanoString panel of human 800 miRNA was used to determine the levels of miRNA in MIAMI cells exposed to treatments indicated on the figure. **a** Unsupervised hierarchical clustering of 30 miRNAs whose levels changed in all treatment groups as indicated. Only those miRNAs with a statistically significantly different expression (*p* < 0.05) were used for analysis. Red and green indicate high and low levels of expression, respectively. **b** Example of treatment-specific expression of miRNAs and their target HLA genes from the dataset. **c** Example of treatment-specific expression of miRNAs and their target immunomodulatory genes from the dataset. The treatment and associated arrows are indicated

First, we performed unsupervised clustering analysis of miRNA expression across treatments to determine if miRNA with similar expression patterns are grouped together based on the treatment applied. The results of this analysis are shown in Fig. 3, and they include 30 miRNAs that were found to be differentially expressed across all treatments (*p* < 0.05)—expression of miRNAs was clustered with treatment groups for all but one sample from TX-treated cells (Fig. 3a). We then utilized IPA’s miRNA-mRNA pairing analysis to uncover if treatment-driven miRNA expression contributed to decreases in immunomodulatory and HLA gene mRNA levels observed in cells treated with IFN**-**γ in combination with TX or CQ compared to cells treated with IFN**-**γ alone (Fig. 3b, c). This pairing analysis revealed that several miRNAs (which can bind to and decrease mRNA levels of genes) are common among all treatments (miR-127-3p, miR-1303, miR-100-5p, miR-191-5p, let-7a-5p, miR15b-5p, and miR-1972). Importantly, we also uncovered miRNAs that are specific for two treatments (miR-4712-5p, miR-1197, miR-513a-5p, miR-199a-5p, miR-338-5p, miR-574-3p, miR-26a-5p, miR-542-3p, and miR-3190-3p) and those which were found to be only expressed in cells subjected to one combination treatment (miR-574-5p, miR-544-3p, and miR-485-3p). This indicates that miRNAs also contribute to the regulation of gene expression and that TX or CQ can, in the presence of IFN**-**γ, stimulate expression of additional miRNAs that contribute to decrease of IFN**-**γ-induced increase in mRNA levels. We then validated miRNA targeting of HLA-DOA by transfecting MIAMI cells with either three miRNAs (hsa-miR-127-3p, hsa-miR-1303, and hsa-miR-100-5p) which target HLA-DOA or miRNA mimic negative control. At 48 h post-transfection, total RNA was collected, and HLA-DOA mRNA level was determined by qPCR. The results indicate that HLA-DOA mRNA level is decreased by 50% compared to MIAMI cell transfected with the miRNA mimics (Additional file 5: Figure S5).

### TX or CQ co-treatments alter CD4+ T cell activation and proliferation

**Fig. 4:**
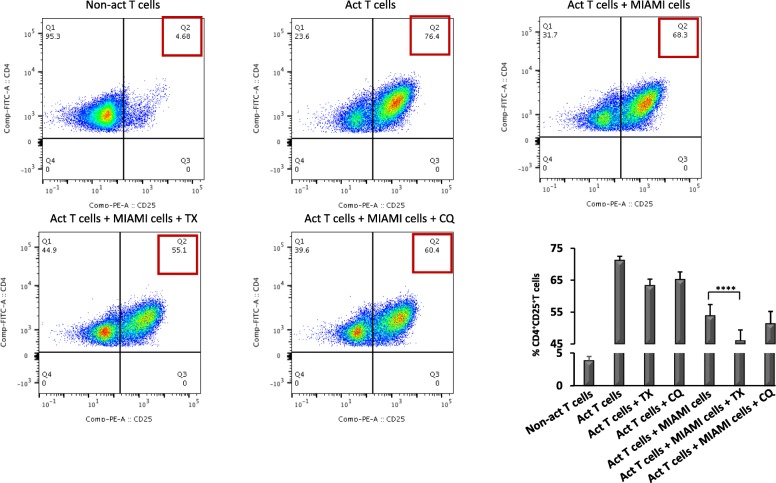
The effects of TX or CQ co-treatments on the reduction of CD25 T cell activation marker. MIAMI cells were co-cultured with activated CD4+ T cells and treated with 5 μM TX or 10 μM CQ for 3 days to evaluate their effect on the expression of the activation marker CD25 on CD4+ T cells. Flow cytometry analysis was used to determine the percentage of CD25+ CD4+ T cells. Graphs are a representation of eight different experiments. “****” *p* ≤ 0.0001

**Fig. 5:**
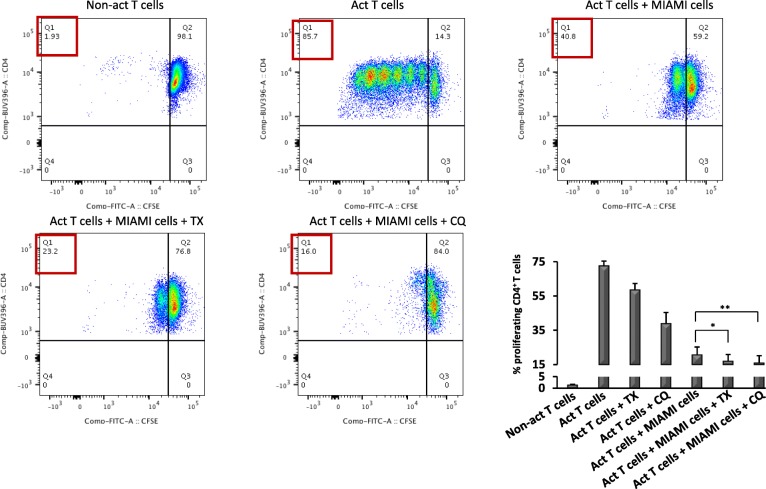
Enhanced suppression of CD4+ T cell proliferation with TX or CQ co-treatments. MIAMI cells were co-cultured with activated CD4+ T cells and treated with 5 μM TX or 10 μM CQ for 4 days to evaluate their effect in causing immunosuppression of CD4+ T cells. Flow cytometry analysis was used to determine the percentage of proliferating CD4+ T cells by CFSE staining. Graphs are a representation of eight different experiments. “*” ≤ 0.05, “**” ≤ 0.01

**Fig. 6:**
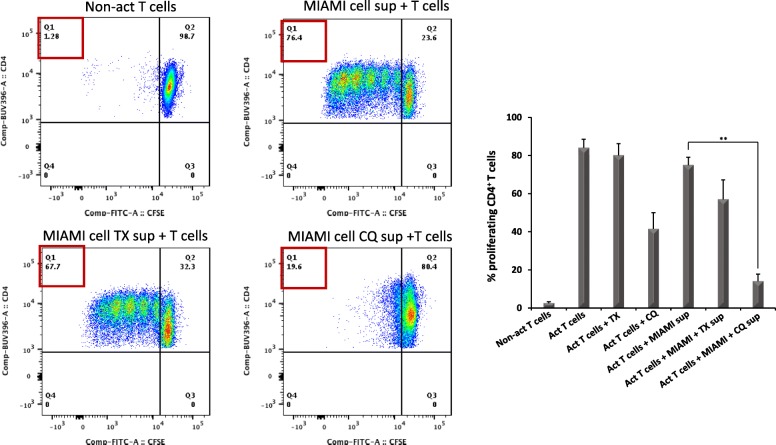
Effect of MIAMI cell supernatants derived from MIAMI cells treated with IFN-γ alone or in combination with TX and CQ on CD4+ T cell proliferation. Miami cells were treated with 500 U/ml IFN-γ alone or in combination with 5 μM TX or 10 μM CQ for 4 days. Supernatants from these treatments were then added to activated CD4+ T cells to evaluate their effects on CD4+ T cell proliferation. Graphs are a representation of three independent experiments; for statistical analysis, we used one-way ANOVA. “**” *p* ≤ 0.01

The results of RNAseq indicate that TX and CQ co-treatments altered mRNA levels of genes involved in MSC-mediated immunomodulation potentially altering responses of activated CD4+ T cells in the presence of MIAMI cells. To evaluate the functional consequences of such changes, CD4+ T cells were isolated from healthy donors (*n* = 8), activated with anti CD3/CD28 antibodies and co-cultured with MIAMI cells in the presence of either CQ or TX for 3 or 4 days. T cell activation was measured by assessing CD25 surface expression and cell proliferation by flow cytometry (gating strategy is presented on Additional file 6: Figure S6). Activated CD4+ T cells co-cultured with MIAMI cells displayed ~ 15% less CD25 expression, but addition of TX or CQ further decreased this expression an additional 10% (*p* < 0.001) or 5% (*p* < 0.05), respectively (Fig. 4). While CD4+ T cell treatment with TX or CQ alone also led to slight decreases in CD25 expression, this decrease was highest in the presence of MIAMI cells. We next evaluated the effects of MIAMI cells CD4+ T cell proliferation in the presence or absence of TX or CQ by staining of CD4+ T cells with CFSE followed by flow cytometry-based analysis on day 4. Our results indicate that addition of either TX or CQ reduced CD4+ T cell proliferation an additional 5–10% compared to MIAMI cells alone (TX, *p* < 0.01; CQ, *p* < 0.001) (Fig. 5). Similarly, to what was observed in the T cell activation experiments, both TX and CQ affected T cell proliferation alone, but this effect was enhanced when the two drugs were combined with MIAMI cells. This indicates that TX and CQ further decrease MIAMI cell-mediated suppression of CD4+ T cells by reducing the level of activation and proliferation. Next, in order to evaluate whether the co-treatments had an effect on MIAMI cell’s secretome, we treated MIAMI cells with TX (5 μM) or CQ (10 μM) in combination with IFN-γ (500 U/ml) for a period of 4 days. As controls, we incubated media with TX and CQ in combination of IFN-γ but without MIAMI cells. Supernatants from these treatments were collected and then used to assess activated CD4 T cell proliferation as described above. Our results indicate that both TX and CQ influence paracrine secretion of MIAMI cells to enhance suppression of T cell proliferation (Fig. 6) compared to vehicle-treated MIAMI cell secretome. This effect on T cell proliferation was more pronounced when T cells were exposed to secretome of MIAMI cells treated with CQ for 4 days (approximately 5-fold enhanced suppression). In contrast, less effect was observed when activated T cells were exposed to conditioned media of MIAMI cells treated with TX.

### TX or CQ co-treatments further increase expression of the programmed death ligand-1

**Fig. 7:**
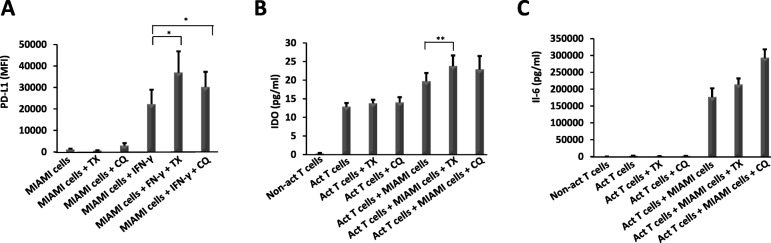
TX co-treatment increases both PDL-1 expression and indoleamine 2,3-dioxygenase secretion. MIAMI cells were treated with IFN-γ with or without 5 μM TX or 10 μM CQ for 4 days to evaluate the effect of the treatments on expression of PD-L1 protein using flow cytometry analysis. Graphs are a representation of six independent experiments (**a**). MIAMI cells were co-cultured with activated CD4+ T cells in the presence of 5 μM TX or 10 μM CQ for 3 days to determine IDO expression in culture supernatants. ELISA analysis in culture supernatants was used to quantify IDO secretion (**b**), and IL-6 ELISA analysis in culture supernatants was used to quantify IL-6 secretion (**c**). “*” ≤ 0.05, “**” ≤ 0.01

Programmed death ligand-1 (PD-L1), a type 1 transmembrane protein, plays an important role in suppressing the immune response by binding to programmed death-1 (PD-1) receptor on T cells. PD-1’s mechanism of action is to suppress the activation of T cells causing immune tolerance or a non-responsive T cell immunity [32]. It was shown previously that exposing MSCs to inflammation upregulates surface expression of PD-L1 which contributes to MSC-mediated immunomodulation [33]. Our RNAseq data identified increased mRNA for PD-L1 which were further upregulated by CQ or TX treatments. To determine whether this increase in mRNA levels also translates to increases in protein levels, we treated MIAMI cells with 500 U/ml IFN**-**γ in combination with 5 μM TX or 10 μM CQ for 4 days followed by assessment of PD-L1 protein expression levels by flow cytometry. MIAMI cells exposed to IFN-γ and co-treated with TX or CQ significantly upregulated the expression of PD-L1 (*p* ≤ 0.05) compared to IFN**-**γ alone (Fig. 7a). This indicates that TX and CQ further upregulate surface expression of PD-L1 potentially increasing MSC-mediated immunotolerance [32].

### Co-treatments increase indoleamine 2,3-dyoxygenase and IL-6 levels

Indoleamine 2,3-dyoxygenase (IDO) is a strong suppressor of immune response by degrading extracellular tryptophan thus reducing T cell proliferation and inflammation [34]. IDO is secreted by multiple cell types (including MSCs) after exposure to inflammatory cytokines like IFN-γ [35]. To determine whether TX or CQ co-treatments affected IDO secretion in co-cultures of MIAMI and CD4+ T cells, we collected the supernatants from co-culture experiments (above) and measured IDO protein levels by ELISA. The data indicate that addition of TX in co-culture assays significantly enhanced IDO secretion compared to CQ co-treatment or MIAMI-T cells-only co-culture (Fig. 7b). On the other hand, CQ co-treatment also increased IDO levels, but this increase was not found to be statistically significant. This indicates that co-treatment with TX enhances the expression of IDO. We also evaluated the levels of IL-6 because of its importance in MSC maintenance and cell stemness [36] and its relationship with anti-inflammatory protective effect in graft-versus-host disease [37, 38]. Our results showed that co-treatment with TX or CQ increases IL-6 secretion, and while we did not find it statistically significant (*n* = 4), we clearly observed an effect.

Altogether, our results from co-culture experiments indicate that overall, TX is more potent in enhancing MIAMI cell’s ability to downregulate responses of activated CD4+ T cell in vitro.

## Discussion

MSCs, adult stem/stromal cells, can be used clinically for regeneration of tissues damaged with prolonged and/or excessive inflammation due to MSCs’ ability to “sense” inflammation and subsequently adopt an anti-inflammatory phenotype [39–41]. While initial pre- and clinical trials indicated relatively modest but promising therapeutic efficacy of MSCs, little was done to evaluate approaches that can be used to increase cells’ therapeutic efficacy. Namely, pre- and clinical studies have shown that the majority of injected MSCs are cleared from the body within several days and multiple injections of low-passage MSCs are required for improved clinical outcomes. Therefore, novel approaches, including combination therapies, that increase MSC survival are needed to increase their therapeutic efficacy. A potential way to achieve this would be to use combinational therapies that target some key aspects of MSC biology. For example, stimulation of pathways that extend cell survival while facilitating maintenance of injected cells would be beneficial for the extended survival of MSCs. One such pathway is autophagy—its upregulation is typically seen in stressed cells during nutrient depravation. However, autophagy also facilitates cell survival during other types of toxic insults and is involved in regulation of inflammation in part by effecting the transcription, processing, and secretion of several cytokines, as well as being regulated by cytokines. Therefore, therapeutic modulation of autophagy might not only extend the life of MSCs but also change how MSCs respond to inflammation.

We hypothesized that modulation of autophagy with small molecules will affect MSCs’ response to inflammation leading to changes in their immunoregulatory properties. To test this hypothesis, we exposed MSC precursor (MIAMI) cells to two pharmacological modulators of autophagy, stimulator TX and inhibitor CQ, in the presence of IFN**-**γ. We opted to use MIAMI cells, which are MSC precursor cells, due to their more primitive stage and higher differentiation potential compared to BM-MSCs [22, 26]. We show that the two drugs altered mRNA levels of genes whose expression was upregulated due to inflammation stimulated by IFN**-**γ. Furthermore, we also show that stimulation and, to a lesser extent, inhibition of autophagy also enhanced the ability of the cells to downregulate responses of activated CD4+ T cells in vitro.

Studies have consistently shown that the pro-inflammatory microenvironment is an important factor for stimulation of MSCs’ immunomodulatory properties. In the presence of cytokines, MSCs upregulate antigen presentation genes without concomitant upregulation of co-stimulatory molecules, resulting in anergy of activated T cells. Additionally, in a pro-inflammatory milieu, MSCs also secrete molecules such as PTGS2, NOS2, IL-10, and/or TGF-β, leading to enhancement of immunomodulation [42, 43]. Our gene expression analysis revealed that the addition of TX or CQ decreased mRNA levels of multiple antigen presentation genes compared to cells treated with IFN**-**γ. Furthermore, TX or CQ also enhanced the expression of immunoregulatory molecules such as IDO1 and PD-L1 compared to IFN**-**γ alone. We also confirmed the effects of TX and CQ on mRNA levels of a number of genes by NanoString gene expression analysis using two different MIAMI cell donors, as well as Ad-MSCs (additional data). These data indicate that TX or CQ have a similar effect on mRNA levels of genes responsible for MSC immunomodulatory properties.

In addition to changes in mRNA levels, we found that TX or CQ co-treatments led to a further increase in surface expression and/or secretion of proteins involved in immune regulation such as PD-L1 and IDO-1. Using co-culture assays of MIAMI and activated CD4+ T cells, we also demonstrated that CQ or TX co-treatments caused further reduction of the activation marker CD25 expression and a significant reduction of CD4+ T cell proliferation compared to vehicle-treated cells. Similar effect was observed when we used supernatants generated by exposing MIAMI cells to CQ and, to much lesser extent, TX, although interpretation of the data is further complicated because CQ alone had a strong suppressive effect on CD4+ T cell proliferation. This CQ-enhanced MIAMI cell secretome-mediated decrease in activated CD4+ T cell proliferation could be explained in part by CQ-mediated stimulation of exosome secretion observed in other cells [44], and MSC-derived exosomes have been shown to possess immunomodulatory properties [45, 46]. Altogether, these results suggested that both TX and CQ enhance immunoregulatory capacity of MIAMI cells in vitro via multiple parallel mechanisms.

Several other studies have also examined the effect of autophagy stimulators/inhibitors on MSCs. Interestingly, such studies reported discrepancies regarding the effects of autophagy stimulators/inhibitors on the therapeutic properties of BM-MSCs. Gao et al. found that stimulation of autophagy with rapamycin enhanced BM-MSC-mediated immunomodulation via enhanced secretion of TGF-β, whereas inhibition with 3-methyladenine weakened their ability to decrease T cell proliferation [47]. On the other hand, using an experimental autoimmune encephalomyelitis model, Dang et al. found that genetic or pharmacological inhibition of autophagy increases the immunomodulatory properties of BM-MSCs by increasing the generation of reactive oxygen species and the activation of kinase essential for prostaglandin E2 expression [42]. Moreover, Wang et al. showed that stimulation of autophagy using rapamycin enhanced the immunosuppressive properties of BM-MSCs which also depended on the duration of rapamycin exposure, with short-term pre-treatment increasing this suppression but long-term treatments showing no effects [48]. These discrepancies highlight the need to carefully interpret data with respect to approaches used to activate/inhibit autophagy but also to consider a possibility that different types of MSCs may differ in their response to inhibition/stimulation. Also, the use of a pharmacological drug to manipulate specific pathways always carries the risk of the activation or inhibition of other non-specific responses. However, pharmacological targeting of MSCs using existing FDA-approved drugs may represent a rapid way to improve their immunoregulatory properties without the high cost and lengthy approval process that is required to utilize genetically modified MSCs in clinics.

## Conclusion

In summary, our data indicate that MIAMI cells’ immunoregulatory properties could be enhanced in vitro using FDA-approved drugs that are inducers and inhibitors of autophagy. This suggests that pharmacologic manipulation of autophagy in MSCs can be potentially used in clinics to achieve better therapeutic outcomes.

## Supplementary information

**Additional file 1: Figure S1.** TX or CQ treatments do not cause significant cytotoxicity or apoptosis but cause accumulation of autophagic vehicles in MIAMI cells. The cells were treated as indicated on panels for 4 days to evaluate apoptosis and induction of autophagic vehicles. (A) light microscopy assessment of cells (left) upon treatment; Annexin V and 7-AAD staining of MIAMI cells after exposure to TX or CQ (right panels). Staurosporine was used as a positive control. (B) Autophagosomes were assessed by examining LC3B levels using immunoblotting and semi-quantified using ImageJ. (C) Assessment of autophagic vehicles using Cyto-ID staining and flow cytometry analysis. The intensity of staining is expressed as mean fluorescence analysis (lower panel). Means ± SEMs (error bars) are shown.

**Additional file 2: Figure S2.** Gene set enrichment analysis for the three distinct treatments as indicated on top. Biological processes, Cellular components and Molecular functions are indicated on the left and the number of genes belonging to a particular category is indicated next to the bar.

**Additional file 3: Figure S3.** Pathway networks for the three distinct treatments. A) IFN-γ; B) IFN-γ + TX; C) IFN-γ + CQ. The lines which connect pathways have numbers of common genes indicated next to them.

**Additional file 4: Figure S4.** NanoString assessment of selected HLA gene expression. MIAMI cells were treated with IFN-γ (blue bars), IFN-γ + CQ (red bars) or IFN-γ + TX (gray bars). Validation of RNA sequencing data was performed for selected genes using MIAMI cell donor 3515 (A), while donor 4381 (B) and adipose-derived MSCs (C) were used for comparison.

**Additional file 5: Figure S5.** MIAMI cells transfected with miRNA mimics were assessed for differences in mRNA levels of HLA-DOA by qPCR. Results were expressed as fold induction compared to the miRNA mimic negative control.

**Additional file 6: Figure S6.** Flow cytometry gating strategy. T cells were stained with Live/dead stain to exclude dead cells in all our experiments unless stated otherwise. (A) Gating strategy for assessment of activated T cells; (B) Gating strategy for assessing T cell proliferation.

## Data Availability

The data materials supporting the current study are included within the article and additional files.

## Abbreviations

7-AAD: 7-Aminoactinomycin D
Ad-MSCs: Adipose-derived MSCs
BM-MSCs: Bone marrow MSCs
CFSE: Carboxylfluorescein succinimidyl ester
CQ: Chloroquine
DMEM: Dulbecco’s modified Eagle’s medium
ELISA: Enzyme-linked immunosorbent assay
ESCs: Embryonic stem cells
FDA: Food and Drug Administration
FDR: False discovery rate
GPRs: General purpose reagents
GVHD: Graft-versus-host disease
HLA: Human leukocyte antigen
IDO: Indoleamine 2,3-dyoxygenase
IFN-γ: Interferon gamma
IL: Interleukin
IPA: Ingenuity pathway analysis
LC3: Microtubule-associated protein light chain
MHC: Major histocompatibility complex
MIAMI: Marrow-isolated adult multilineage
miRNA: Micro RNA
mRNA: Messenger RNA
MSCs: Mesenchymal stromal cells
NOS2: Nitric oxide synthase 2
PBMC: Peripheral blood mononuclear cells
PD-L1: Programmed death ligand-1
PTGS2: Prostaglandin-endoperoxide synthase 2
RNA-seq: RNA sequencing
TGF-β: Transforming growth factor beta
TX: Tamoxifen
