# Supplementary material for: Combination therapies enhance immunoregulatory properties of MIAMI cells
Source: Stem Cell Res Ther. 2019 Dec 18;10:395. doi: 10.1186/s13287-019-1515-3 (PMC6921447; doi:10.1186/s13287-019-1515-3)
Supplement: Supplementary file 1 — Additional file 1: Figure S1. TX or CQ treatments do not cause significant cytotoxicity or apoptosis but cause accumulation of autophagic vehicles in MIAMI cells. The cells were treated as indicated on panels for 4 days to evaluate apoptosis and induction of autophagic vehicles. (A) light microscopy assessment of cells (left) upon treatment; Annexin V and 7-AAD staining of MIAMI cells after exposure to TX or CQ (right panels). Staurosporine was used as a positive control. (B) Autophagosomes were assessed by examining LC3B levels using immunoblotting and semi-quantified using ImageJ. (C) Assessment of autophagic vehicles using Cyto-ID staining and flow cytometry analysis. The intensity of staining is expressed as mean fluorescence analysis (lower panel). Means ± SEMs (error bars) are shown. [file 13287_2019_1515_MOESM1_ESM.pptx]

## Slide 1
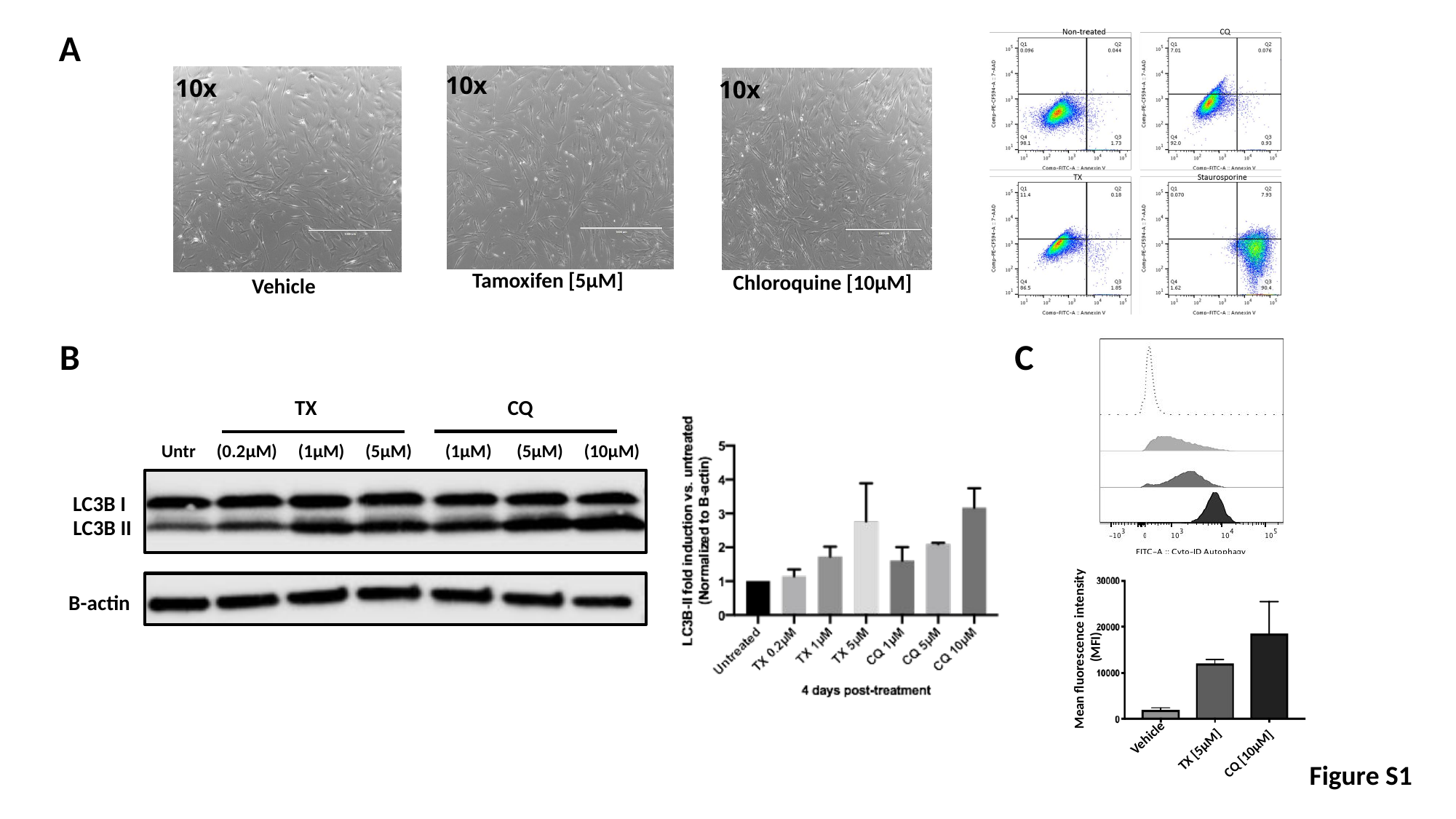

A
10x
10x
10x
Tamoxifen [5µM]
Chloroquine [10µM]
Vehicle
C
B
CQ
TX
 Untr (0.2µM) (1µM) (5µM) (1µM) (5µM) (10µM)
LC3B I
LC3B II
B-actin
Mean fluorescence intensity
(MFI)
Vehicle
TX [5µM]
CQ [10µM]
Figure S1
