# Supplementary figures and images for: Combination therapies enhance immunoregulatory properties of MIAMI cells
Source: Stem Cell Res Ther. 2019 Dec 18;10:395. doi: 10.1186/s13287-019-1515-3 (PMC6921447; doi:10.1186/s13287-019-1515-3)

## Slide 1
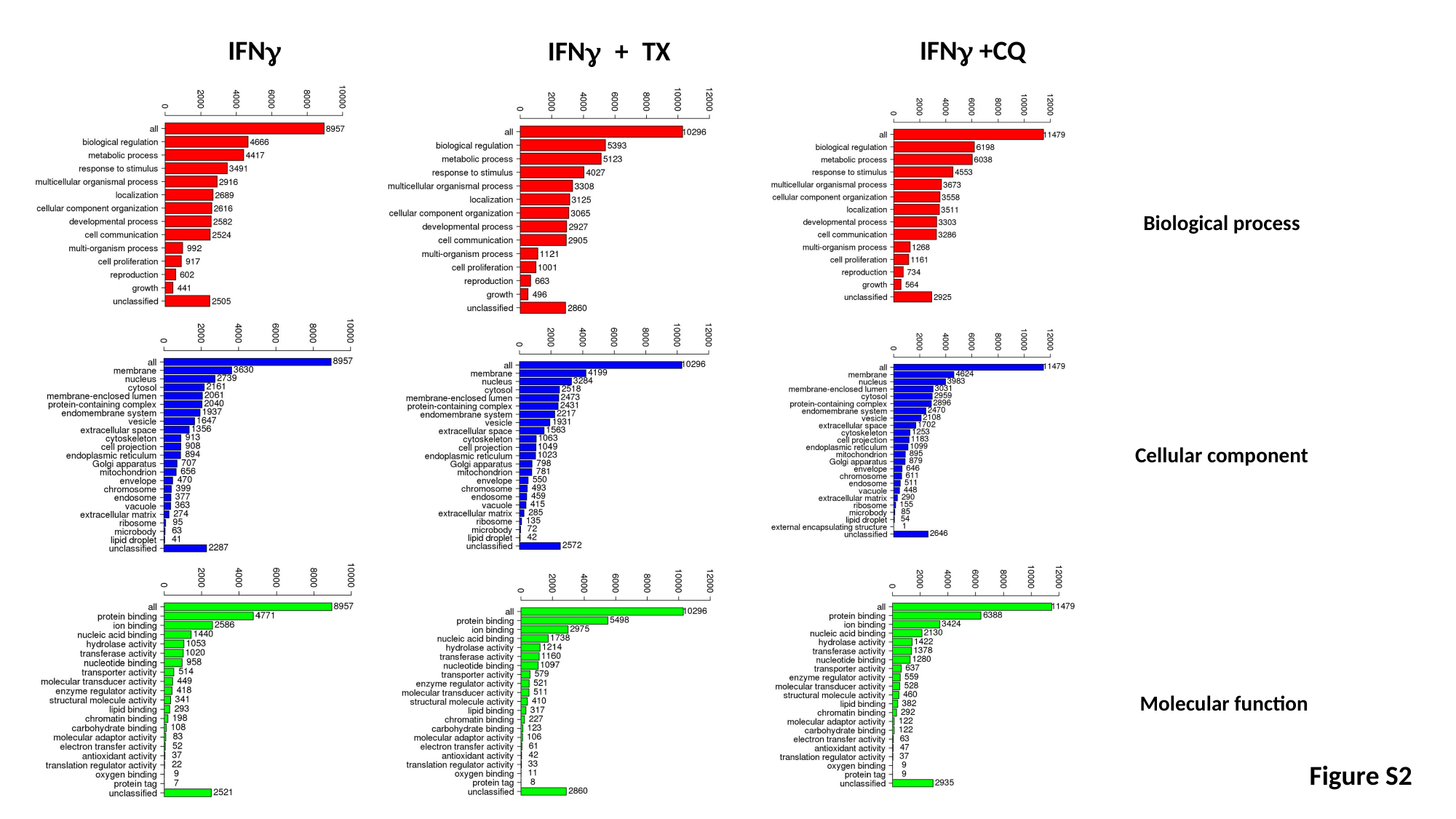

IFNg
IFNg +CQ
IFNg + TX
Biological process
Cellular component
Molecular function
Figure S2

Supplement: Supplementary file 2 — Additional file 2: Figure S2. Gene set enrichment analysis for the three distinct treatments as indicated on top. Biological processes, Cellular components and Molecular functions are indicated on the left and the number of genes belonging to a particular category is indicated next to the bar. [file 13287_2019_1515_MOESM2_ESM.pptx]

## Slide 1
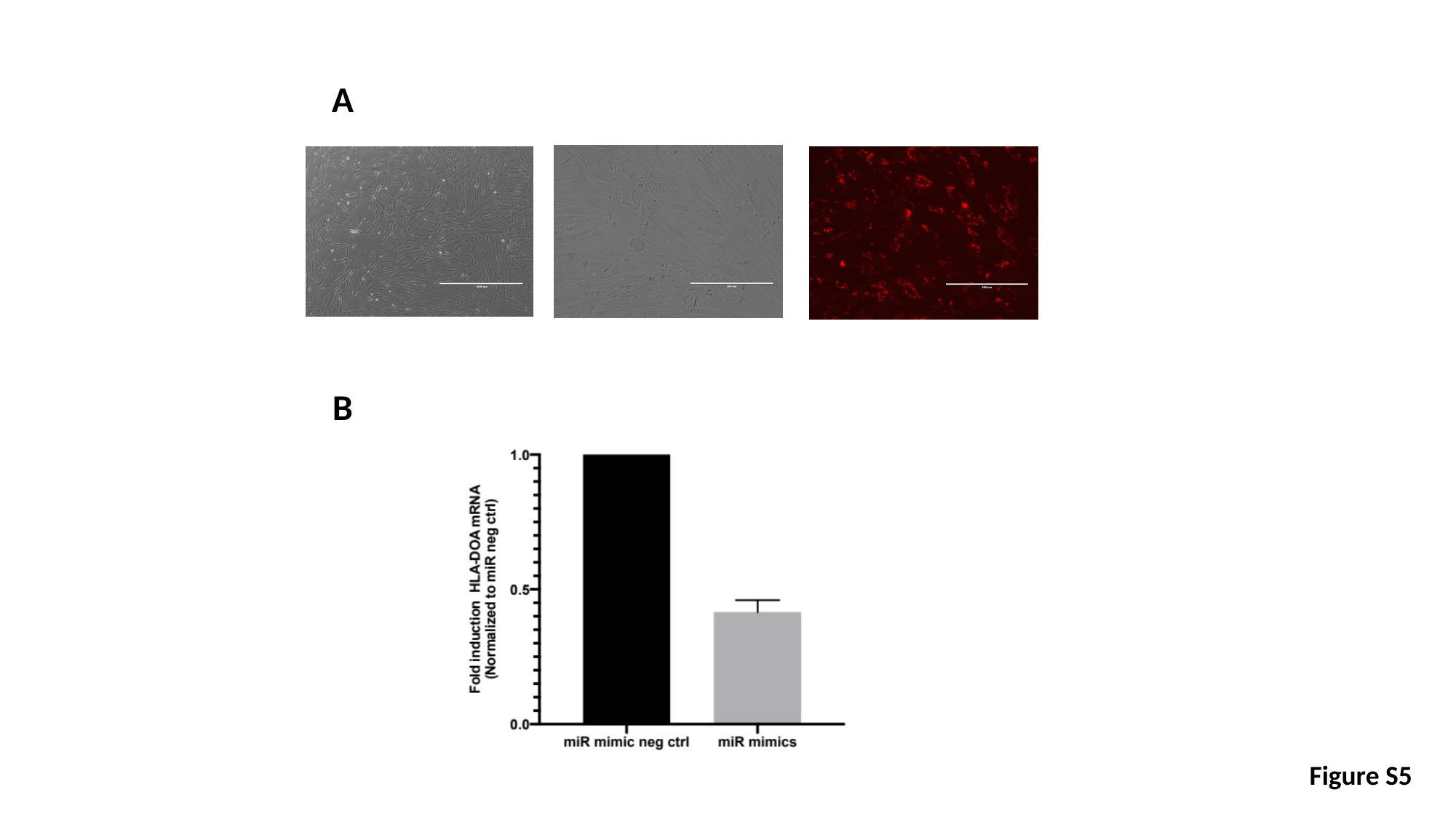

A
B
Figure S5

Supplement: Supplementary file 5 — Additional file 5: Figure S5. MIAMI cells transfected with miRNA mimics were assessed for differences in mRNA levels of HLA-DOA by qPCR. Results were expressed as fold induction compared to the miRNA mimic negative control. [file 13287_2019_1515_MOESM5_ESM.pptx]

## Slide 1
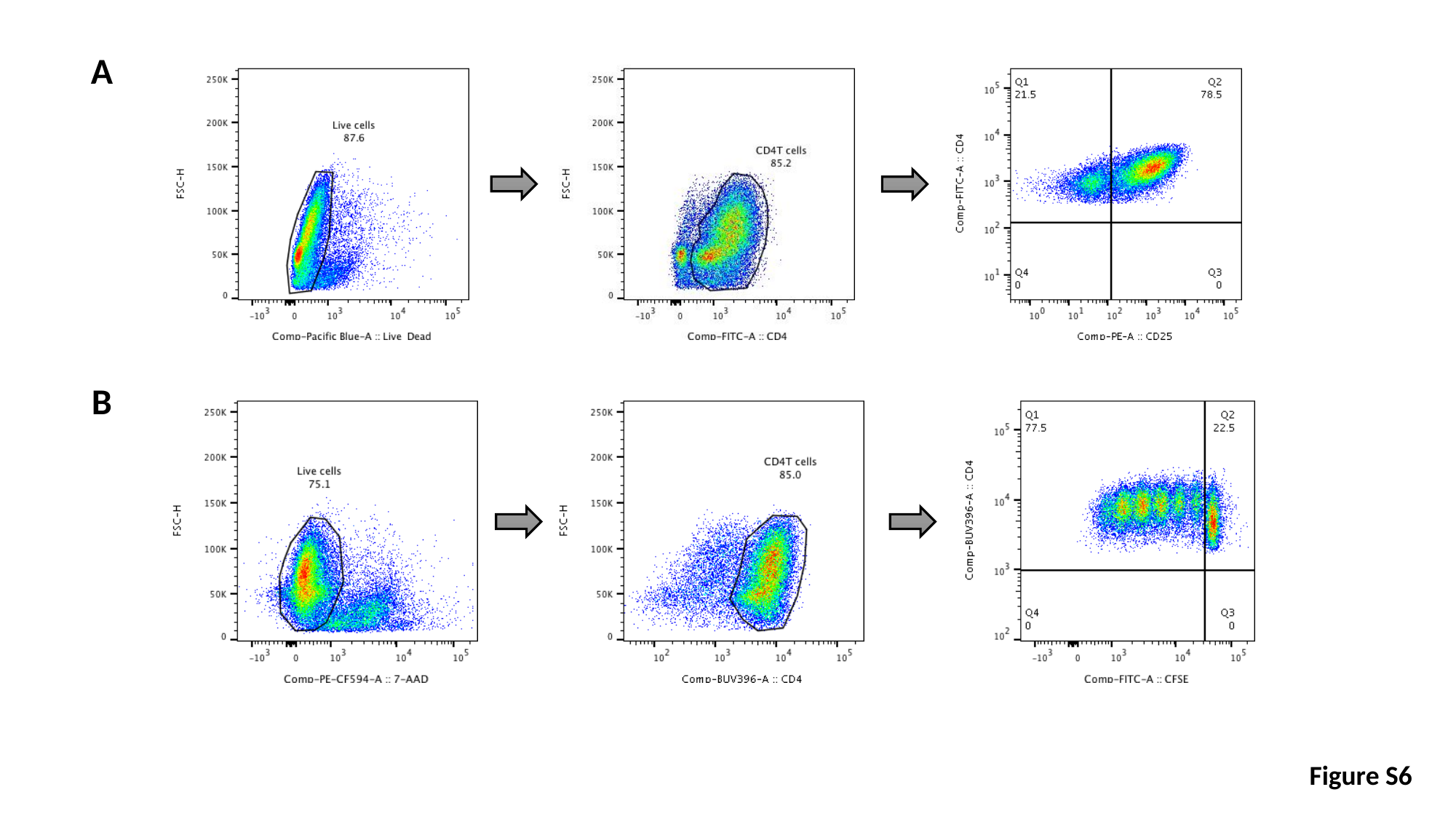

A
B
Figure S6

Supplement: Supplementary file 6 — Additional file 6: Figure S6. Flow cytometry gating strategy. T cells were stained with Live/dead stain to exclude dead cells in all our experiments unless stated otherwise. (A) Gating strategy for assessment of activated T cells; (B) Gating strategy for assessing T cell proliferation. [file 13287_2019_1515_MOESM6_ESM.pptx]
