# Supplementary material for: Combination therapies enhance immunoregulatory properties of MIAMI cells
Source: Stem Cell Res Ther. 2019 Dec 18;10:395. doi: 10.1186/s13287-019-1515-3 (PMC6921447; doi:10.1186/s13287-019-1515-3)
Supplement: Supplementary file 3 — Additional file 3: Figure S3. Pathway networks for the three distinct treatments. A) IFN-γ; B) IFN-γ + TX; C) IFN-γ + CQ. The lines which connect pathways have numbers of common genes indicated next to them. [file 13287_2019_1515_MOESM3_ESM.pptx]

## Slide 1
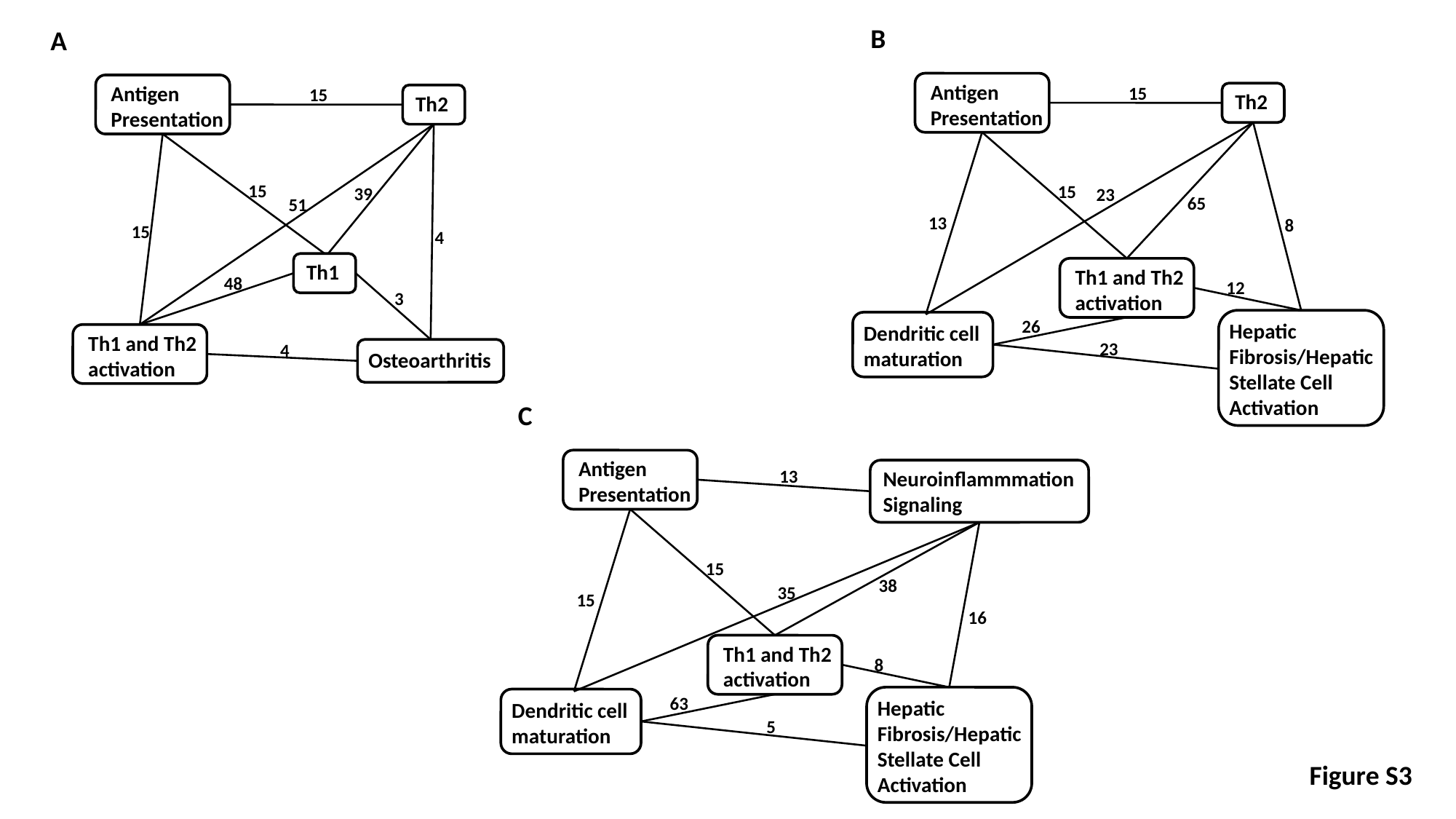

B
Antigen Presentation
15
Th2
15
23
65
13
8
Th1 and Th2 activation
12
26
Hepatic Fibrosis/Hepatic Stellate Cell Activation
Dendritic cell maturation
23
A
Antigen Presentation
15
Th2
15
39
51
15
4
Th1
48
3
Th1 and Th2 activation
4
Osteoarthritis
C
Antigen Presentation
13
Neuroinflammmation Signaling
15
38
35
15
16
Th1 and Th2 activation
8
63
Hepatic Fibrosis/Hepatic Stellate Cell Activation
Dendritic cell maturation
5
Figure S3
