# Supplementary material for: Combination therapies enhance immunoregulatory properties of MIAMI cells
Source: Stem Cell Res Ther. 2019 Dec 18;10:395. doi: 10.1186/s13287-019-1515-3 (PMC6921447; doi:10.1186/s13287-019-1515-3)
Supplement: Supplementary file 4 — Additional file 4: Figure S4. NanoString assessment of selected HLA gene expression. MIAMI cells were treated with IFN-γ (blue bars), IFN-γ + CQ (red bars) or IFN-γ + TX (gray bars). Validation of RNA sequencing data was performed for selected genes using MIAMI cell donor 3515 (A), while donor 4381 (B) and adipose-derived MSCs (C) were used for comparison. [file 13287_2019_1515_MOESM4_ESM.pptx]

## Slide 1
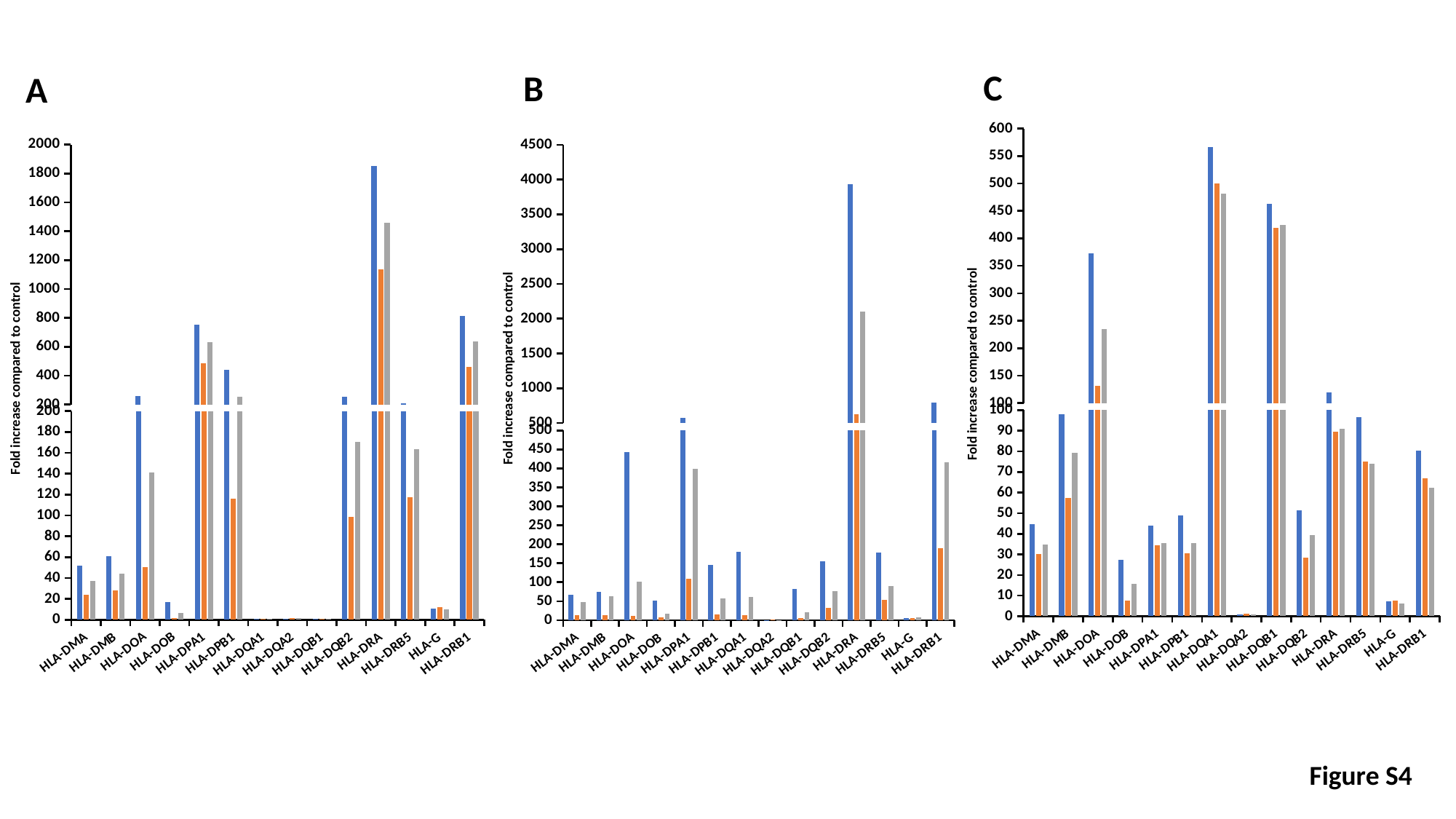

C
B
A
### Chart
| Category | 500 U/ml IFN-g | IFN-g + CQ | IFN-g + TX |
|---|---|---|---|
| HLA-DMA | 44.6722440944882 | 30.22387946698971 | 34.63802241066021 |
| HLA-DMB | 98.17418426103647 | 57.41698656429939 | 79.4203454894434 |
| HLA-DOA | 373.1704067321177 | 131.2026647966339 | 235.3625525946704 |
| HLA-DOB | 27.29871794871793 | 7.451923076923077 | 15.8301282051282 |
| HLA-DPA1 | 43.85472507787119 | 34.5271981070278 | 35.6280363087042 |
| HLA-DPB1 | 48.86180074587108 | 30.47123068726691 | 35.56728822589236 |
| HLA-DQA1 | 566.5916988416989 | 499.5666023166023 | 481.466216216216 |
| HLA-DQA2 | 1.008495145631068 | 1.052184466019417 | 0.870145631067961 |
| HLA-DQB1 | 463.1316239316239 | 419.442735042735 | 425.0931623931625 |
| HLA-DQB2 | 51.57422559906486 | 28.48918760958502 | 39.45616598480421 |
| HLA-DRA | 119.4570345408593 | 89.39929362970643 | 90.81942194284235 |
| HLA-DRB5 | 96.52992398064961 | 74.9326883206634 | 73.88555632342774 |
| HLA-G | 7.156079217196687 | 7.569236974964868 | 6.21622078800812 |
| HLA-DRB1 | 80.49547656035821 | 67.01433184075051 | 62.22078040756647 |
### Chart
| Category | 500 U/ml IFN-g | IFN-g + CQ | IFN-g + TX |
|---|---|---|---|
| HLA-DMA | 44.6722440944882 | 30.22387946698971 | 34.63802241066021 |
| HLA-DMB | 98.17418426103647 | 57.41698656429939 | 79.4203454894434 |
| HLA-DOA | 373.1704067321177 | 131.2026647966339 | 235.3625525946704 |
| HLA-DOB | 27.29871794871793 | 7.451923076923077 | 15.8301282051282 |
| HLA-DPA1 | 43.85472507787119 | 34.5271981070278 | 35.6280363087042 |
| HLA-DPB1 | 48.86180074587108 | 30.47123068726691 | 35.56728822589236 |
| HLA-DQA1 | 566.5916988416989 | 499.5666023166023 | 481.466216216216 |
| HLA-DQA2 | 1.008495145631068 | 1.052184466019417 | 0.870145631067961 |
| HLA-DQB1 | 463.1316239316239 | 419.442735042735 | 425.0931623931625 |
| HLA-DQB2 | 51.57422559906486 | 28.48918760958502 | 39.45616598480421 |
| HLA-DRA | 119.4570345408593 | 89.39929362970643 | 90.81942194284235 |
| HLA-DRB5 | 96.52992398064961 | 74.9326883206634 | 73.88555632342774 |
| HLA-G | 7.156079217196687 | 7.569236974964868 | 6.21622078800812 |
| HLA-DRB1 | 80.49547656035821 | 67.01433184075051 | 62.22078040756647 |
### Chart
| Category | 500 U/ml IFN-g | IFN-g + CQ | IFN-g + TX |
|---|---|---|---|
| HLA-DMA | 51.64916245679339 | 23.64477532571125 | 36.84937516617921 |
| HLA-DMB | 60.56731757451181 | 28.27852004110995 | 43.8586844809866 |
| HLA-DOA | 260.1341107871718 | 50.33138969873659 | 141.1195335276968 |
| HLA-DOB | 16.59475218658892 | 1.37220602526725 | 6.360544217687072 |
| HLA-DPA1 | 753.4643449419568 | 485.0356550580429 | 633.8548922056384 |
| HLA-DPB1 | 438.5714285714286 | 116.2691933916424 | 255.1525753158406 |
| HLA-DQA1 | 0.705065926439972 | 1.09437890353921 | 0.596807772380292 |
| HLA-DQA2 | 0.987366375121477 | 1.505344995140913 | 1.528668610301263 |
| HLA-DQB1 | 0.730409777138749 | 1.01509705248023 | 0.618260244428469 |
| HLA-DQB2 | 252.9489867225716 | 98.85499650593988 | 170.2547169811321 |
| HLA-DRA | 1850.190476190476 | 1135.740524781341 | 1457.643343051506 |
| HLA-DRB5 | 210.357628765792 | 117.3187560738581 | 163.4373177842566 |
| HLA-G | 10.37889290012034 | 12.12726835138387 | 10.26471720818291 |
| HLA-DRB1 | 811.8923594425754 | 458.0134550696778 | 637.1902931283034 |
### Chart
| Category | 500 U/ml IFN-g | IFN-g + CQ | IFN-g + TX |
|---|---|---|---|
| HLA-DMA | 51.64916245679339 | 23.64477532571125 | 36.84937516617921 |
| HLA-DMB | 60.56731757451181 | 28.27852004110995 | 43.8586844809866 |
| HLA-DOA | 260.1341107871718 | 50.33138969873659 | 141.1195335276968 |
| HLA-DOB | 16.59475218658892 | 1.37220602526725 | 6.360544217687072 |
| HLA-DPA1 | 753.4643449419568 | 485.0356550580429 | 633.8548922056384 |
| HLA-DPB1 | 438.5714285714286 | 116.2691933916424 | 255.1525753158406 |
| HLA-DQA1 | 0.705065926439972 | 1.09437890353921 | 0.596807772380292 |
| HLA-DQA2 | 0.987366375121477 | 1.505344995140913 | 1.528668610301263 |
| HLA-DQB1 | 0.730409777138749 | 1.01509705248023 | 0.618260244428469 |
| HLA-DQB2 | 252.9489867225716 | 98.85499650593988 | 170.2547169811321 |
| HLA-DRA | 1850.190476190476 | 1135.740524781341 | 1457.643343051506 |
| HLA-DRB5 | 210.357628765792 | 117.3187560738581 | 163.4373177842566 |
| HLA-G | 10.37889290012034 | 12.12726835138387 | 10.26471720818291 |
| HLA-DRB1 | 811.8923594425754 | 458.0134550696778 | 637.1902931283034 |
### Chart
| Category | 500 U/ml IFN-g | IFN-g + CQ | IFN-g + TX |
|---|---|---|---|
| HLA-DMA | 67.93024309225427 | 12.38924958478032 | 47.42624188434242 |
| HLA-DMB | 73.87258912916417 | 12.5499707773232 | 63.68147282291058 |
| HLA-DOA | 443.7430555555556 | 11.93402777777777 | 102.0052083333333 |
| HLA-DOB | 52.46875000000001 | 7.003472222222223 | 17.43055555555556 |
| HLA-DPA1 | 572.1089108910892 | 109.503300330033 | 398.8787128712869 |
| HLA-DPB1 | 145.1002189248358 | 15.0058379956215 | 56.54974458769153 |
| HLA-DQA1 | 180.6182965299684 | 14.2429022082019 | 60.8738170347003 |
| HLA-DQA2 | 0.582128777923785 | 1.086727989487517 | 0.565045992115637 |
| HLA-DQB1 | 83.21233974358971 | 5.880608974358975 | 21.33092948717949 |
| HLA-DQB2 | 154.6277030976038 | 32.34482758620688 | 76.90356516656925 |
| HLA-DRA | 3939.649305555556 | 620.6631944444445 | 2105.897569444445 |
| HLA-DRB5 | 177.353470437018 | 54.2827763496144 | 90.09383033419022 |
| HLA-G | 6.666814266162141 | 5.636502697045327 | 6.794856828489386 |
| HLA-DRB1 | 794.800313643492 | 190.7626764244642 | 415.662310507057 |
### Chart
| Category | 500 U/ml IFN-g | IFN-g + CQ | IFN-g + TX |
|---|---|---|---|
| HLA-DMA | 67.93024309225427 | 12.38924958478032 | 47.42624188434242 |
| HLA-DMB | 73.87258912916417 | 12.5499707773232 | 63.68147282291058 |
| HLA-DOA | 443.7430555555556 | 11.93402777777777 | 102.0052083333333 |
| HLA-DOB | 52.46875000000001 | 7.003472222222223 | 17.43055555555556 |
| HLA-DPA1 | 572.1089108910892 | 109.503300330033 | 398.8787128712869 |
| HLA-DPB1 | 145.1002189248358 | 15.0058379956215 | 56.54974458769153 |
| HLA-DQA1 | 180.6182965299684 | 14.2429022082019 | 60.8738170347003 |
| HLA-DQA2 | 0.582128777923785 | 1.086727989487517 | 0.565045992115637 |
| HLA-DQB1 | 83.21233974358971 | 5.880608974358975 | 21.33092948717949 |
| HLA-DQB2 | 154.6277030976038 | 32.34482758620688 | 76.90356516656925 |
| HLA-DRA | 3939.649305555556 | 620.6631944444445 | 2105.897569444445 |
| HLA-DRB5 | 177.353470437018 | 54.2827763496144 | 90.09383033419022 |
| HLA-G | 6.666814266162141 | 5.636502697045327 | 6.794856828489386 |
| HLA-DRB1 | 794.800313643492 | 190.7626764244642 | 415.662310507057 |Fold increase compared to control
Fold increase compared to control
Fold increase compared to control
Figure S4
